# Ethical Design of Data-Driven Decision Support Tools for Improving Cancer Care: Embedded Ethics Review of the 4D PICTURE Project

**DOI:** 10.2196/65566

**Published:** 2025-04-10

**Authors:** Marieke Bak, Laura Hartman, Charlotte Graafland, Ida J Korfage, Alena Buyx, Maartje Schermer, 4D PICTURE Consortium

**Affiliations:** 1 Institute of History and Ethics in Medicine, Department of Preclinical Medicine TUM School of Medicine and Health Technical University of Munich München Germany; 2 Department of Ethics, Law and Humanities Amsterdam UMC University of Amsterdam Amsterdam The Netherlands; 3 Department of Medical Ethics, Philosophy and History of Medicine Erasmus Medical Center Rotterdam The Netherlands; 4 see Acknowledgments; 5 Erasmus School of Health Policy & Management Rotterdam The Netherlands

**Keywords:** shared decision-making, oncology, IT, ethics, decision support tools, big data, medical decision-making, artificial intelligence

## Abstract

Oncology patients often face complex choices between treatment regimens with different risk-benefit ratios. The 4D PICTURE (Producing Improved Cancer Outcomes Through User-Centered Research) project aims to support patients, their families, and clinicians with these complex decisions by developing data-driven decision support tools (DSTs) for patients with breast cancer, prostate cancer, and melanoma as part of care path redesign using a methodology called MetroMapping. There are myriad ethical issues to consider as the project will create data-driven prognostic models and develop conversation tools using artificial intelligence while including patient perspectives by setting up boards of experiential experts in 8 different countries. This paper aims to review the key ethical challenges related to the design and development of DSTs in oncology. To explore the ethics of DSTs in cancer care, the project adopted the Embedded Ethics approach—embedding ethicists into research teams to sensitize team members to ethical aspects and assist in reflecting on those aspects throughout the project. We conducted what we call an embedded review of the project drawing from key literature on topics related to the different work packages of the 4D PICTURE project, whereas the analysis was an iterative process involving discussions with researchers in the project. Our review identified 13 key ethical challenges related to the development of DSTs and the redesigning of care paths for more personalized cancer care. Several ethical aspects were related to general potential issues of data bias and privacy but prompted specific research questions, for instance, about the inclusion of certain demographic variables in models. Design methodology in the 4D PICTURE project can provide insights related to design justice, a novel consideration in health care DSTs. Ethical points of attention related to health care policy, such as cost-effectiveness, financial sustainability, and environmental impact, were also identified, along with challenges in the research process itself, emphasizing the importance of epistemic justice, the role of embedded ethicists, and psychological safety. This viewpoint highlights ethical aspects previously neglected in the digital health ethics literature and zooms in on real-world challenges in an ongoing project. It underscores the need for researchers and leaders in data-driven medical research projects to address ethical challenges beyond the scientific core of the project. More generally, our tailored review approach provides a model for embedding ethics into large data-driven oncology research projects from the start, which helps ensure that technological innovations are designed and developed in an appropriate and patient-centered manner.

## Introduction

**Figure 1:**
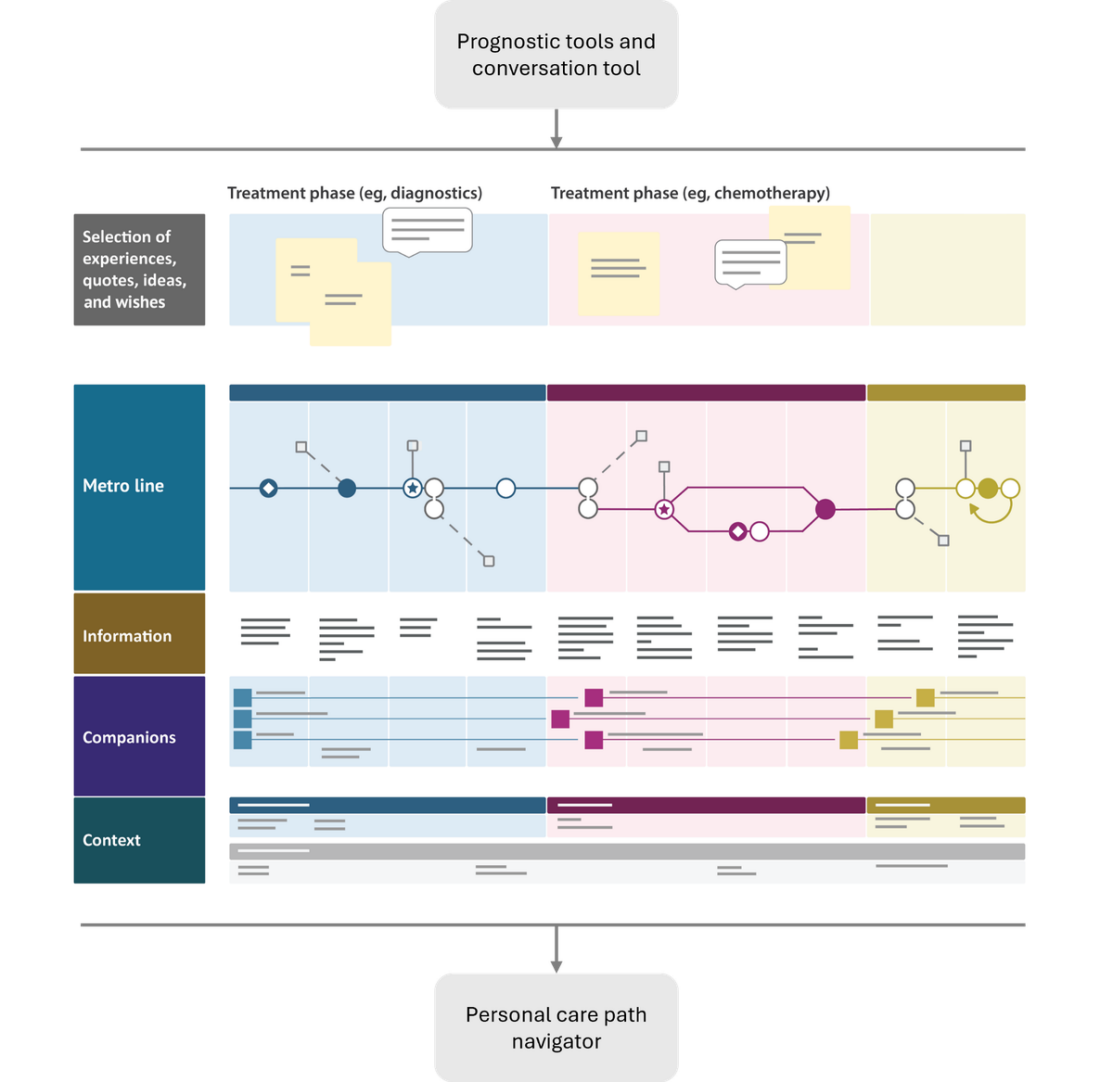
Schematic illustration of the MetroMap that forms the core of the 4D PICTURE (Producing Improved Cancer Outcomes Through User-Centered Research) project. The MetroMap is a comprehensive visualization of the general care trajectory. Feeding into the MetroMap will be the results of two types of models developed in the project: (1) a treatment outcome prediction tool for each cancer type and (2) a conversation tool developed by analyzing patient experiences through text mining. The result of integrating these data-driven tools into the MetroMap will be a personal care path navigator for each patient that serves as a decision aid in shared decision-making.

People diagnosed with cancer often face difficult choices regarding their treatment and potential impact on survival and quality of life [1]. Data-driven decision support tools (DSTs) hold significant potential in empowering patients, enhancing personalized care, and fostering health equity [2,3]. Nevertheless, most current DSTs do not account for individual preferences, which hinders their broader integration into clinical practice. To improve cancer treatment decision-making by addressing existing challenges in DSTs, a large European collaboration was started—the 4D PICTURE (Producing Improved Cancer Outcomes Through User-Centered Research) project [4]. Recognizing the complexity that patients face, the consortium seeks to use design methods (particularly the *MetroMapping* methodology, Figure 1) to improve care paths in oncology. This involves the development of innovative prognostic models and conversation tools that consider patient experiences, values, and preferences through models partly based on artificial intelligence (AI). Collaborating with patients and other stakeholders, the project focuses on breast cancer, prostate cancer, and melanoma, aiming to create comprehensive DSTs for these types of cancer. The use of these tools in the *MetroMap* for redesigning cancer care paths will be evaluated on effectiveness and cost-effectiveness, and social and ethical concerns will be addressed throughout the project.

Ethics is highly relevant to the development of data-driven DSTs for personalizing oncology care. For instance, the use of low-quality prognostic models may lead to incorrect and harmful decisions. When using AI-driven DSTs, concerns about quality are particularly warranted as there is still a lack of robust evidence on their effectiveness [5]. As such, normative principles such as data quality, algorithmic fairness, and data privacy are important to consider when developing data-driven DSTs. However, principles alone cannot guarantee that the developed tools are ethical and acceptable to patients and health care providers [6]. What is needed as well is guidance on how researchers can be practically assisted to anticipate, identify, and address ethical issues of data-driven care based on the specific case at hand. This can be done through the *Embedded Ethics* approach, which stimulates close collaborations between ethicists on the one hand and developers, researchers, and clinicians on the other who work together in an iterative and continuous manner [7]. In the 4D PICTURE project, ethicists are embedded into the project in this way to promote guidance and reflection on the ethics of the entire project.

The first task of the ethics *work package* in a large interdisciplinary project is usually to create an overview of potential ethical challenges that can be expected in that project based on the literature. However, we noticed that such literature surveys often come up with the same general issues. A database search on the ethical aspects of data-driven DSTs in medicine is necessarily broad and will provide high-level findings on the aforementioned principles, which still requires translation to the project at hand to derive actionable recommendations. Moreover, the ethical aspects of such interdisciplinary projects are too heterogenous for a systematic review on the “ethics of data-driven DSTs in healthcare”—ethical questions may also arise in parts of the project not directly related to DST development, such as their evaluation or the dissemination of results. Therefore, in the 4D PICTURE project, we took a different approach. As ethicists in the 4D PICTURE project, we discussed ethics in relation to the different work packages with the project researchers and looked for key publications in the ethics literature on the topics that came up in each work package separately. We moved back and forth between literature and practice in an iterative process to be as specific and close to practice as possible. This resulted in an agenda of aspects that may be ethically relevant within the project and serves as a basis for further empirical and theoretical ethics research.

This viewpoint describes this process and has two interrelated aims as it (1) introduces the *embedded review* approach for identifying ethical aspects within an interdisciplinary research consortium and (2) outlines key ethical challenges to be considered when developing data-driven DSTs for more personalized oncology care. In what follows, we describe our methodology before discussing ethical challenges related to data-driven DSTs in the project under study. This paper provides a detailed overview of the ethics of data-driven DSTs because of the link to a particular project, as well as reference to broader ethical aspects (eg, the ethics of interdisciplinary collaboration or psychological safety in research teams) that are often neglected. We find that this work has relevance beyond the 4D PICTURE project as it is the first review-type paper explicitly grounded in the *Embedded Ethics* approach. Our findings show that, even when simply looking for literature on ethical aspects, a lot can be gained when ethics is embedded into a project from the start.

## Methods

### Embedded Ethics Approach and Study Design

As ethics is about normative argumentation and conceptual analysis, a systematic literature study usually does not suffice for an overview of the ethical literature on a certain topic. Moreover, the ethical aspects of large interdisciplinary projects such as the 4D PICTURE project are too heterogenous for a traditional systematic or scoping review. Therefore, different methods for reviews have been described in the ethics literature suitable for different purposes, ranging from a *rapid review* to a *critical interpretative review* [8]. For the context of this study, our main aim was to sensitize researchers of the consortium to the ethical issues in their work packages and support them with a shared ethical framework, vocabulary, and argumentation to navigate the various ethical issues of the project deliberately and consciously. Our priority was to ensure that the literature was useful, comprehensible, and relevant for the consortium’s needs, thus balancing methodological rigor and depth of analysis with practical applicability. In conducting this review, we aimed to develop directions for specific ethics guidance and further research as well as to “create a shared knowledge base among team members” [9]. Moreover, we wanted to include the ethics *of* the consortium (eg, ensuring psychological safety in research teams) rather than merely looking at the ethics *in* the consortium (eg, avoiding biased outcomes of the prognostic models).

Therefore, we opted for an *embedded review*, which, in the typology of McDougall [8], could be best described as a critical interpretative review, with the main difference that we established the analytical categories together with the researchers of the consortium. This is in line with the paradigm of *Embedded Ethics*, a relatively new approach for integrating ethics into interdisciplinary health care projects that focuses on delivering guidance for practical ethical dilemmas also when these are unexpected and come up ad hoc*.* This is done by embedding ethicists into these projects and stimulating close collaboration so that ethical aspects are taken into account in a continuous and iterative manner [7]. Various tools and methods are used to embed ethics into the development of new technologies; usually, a literature review is the starting point. As Willem et al [9] note, a literature review in an embedded ethics project “provides the opportunity to collectively interrogate the project’s goals.” Thus, the themes described in this paper are based on an iterative approach that we have called an embedded review—going back and forth between research meetings, reading and searching for literature, interactive discussions and meetings with 4D PICTURE researchers, joining trainings, and conducting observations of meetings. Hereafter, we describe how we conducted this embedded review of ethical aspects in the 4D PICTURE project.

### Identification of Ethical Challenges and Member Check

**Figure 2:**
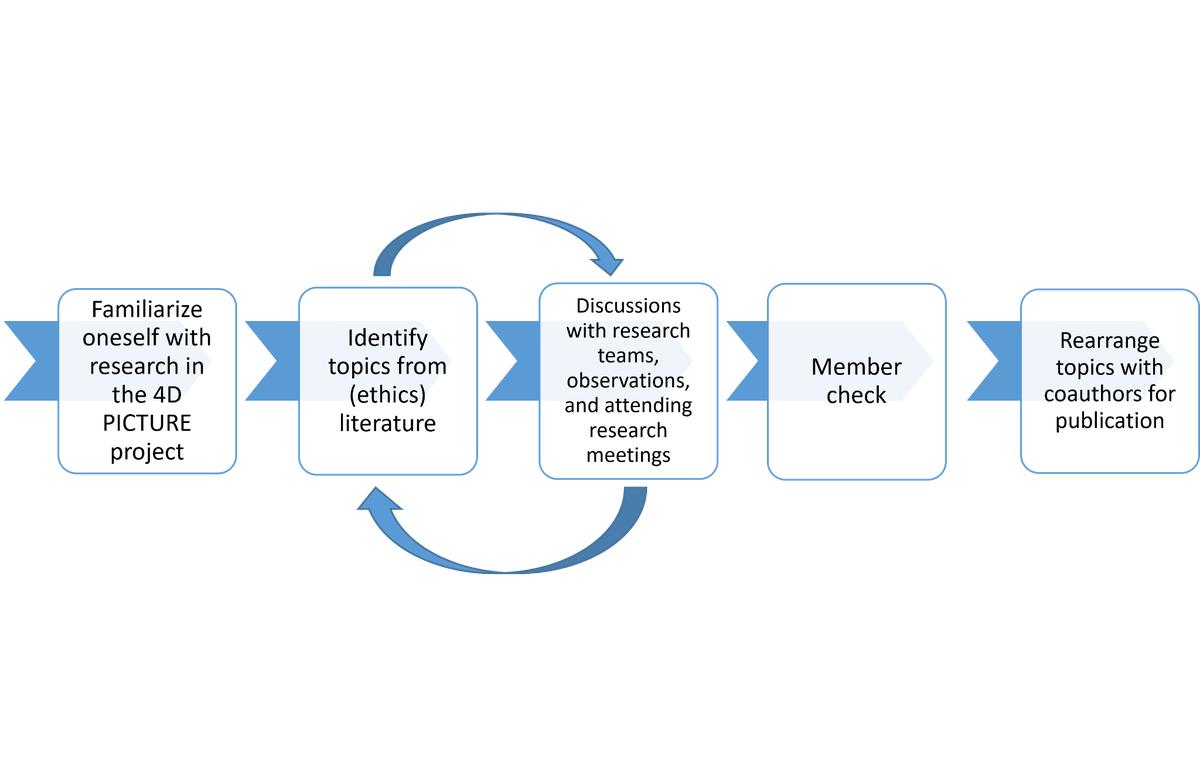
Method of conducting an embedded review in the 4D PICTURE (Producing Improved Cancer Outcomes Through User-Centered Research) project—flowchart of the process of identifying and refining ethical challenges based on the literature and discussions with researchers in the project.

First, 2 authors (LH and CG) familiarized themselves with the research objectives and activities of each work package in the 4D PICTURE project. They identified ethical themes that may be relevant for the activities and research of each work package and looked for key publications in the (empirical and theoretical ethics) literature on that particular topic. Some of these publications were known by the authors, whereas others were found through simple searches in PubMed and Google Scholar as well as through further snowball searches. Of note is that the iterative process of *identifying* did not simply consist of extracting issues from the project proposal or the ethics literature but required active interpretation by the authors to link the literature to the intended work in the various parts of the 4D PICTURE project. After having conducted an initial identification of ethical themes, these insights were summarized in such a way that the descriptions were readable and understandable by researchers without a background in ethics. This document was shared within the consortium and also included a *further reading* section for those interested in reading more. The categories of ethical themes were then further refined through conversations with researchers in the 4D PICTURE project. For instance, the points discussed in the *Results* section about the design of the MetroMap were extensively discussed with researchers at an in-person training in the Netherlands by LH and CG. Finally, 2 of the ethicists in the project (LH and MB) presented the findings of this embedded review during a general meeting that was attended by all the researchers of the 4D PICTURE consortium, which took place in 2023 and served as a *member check* to see whether relevant issues were included but also to stimulate researchers in the project to think about ethics in their work package. An overview of these methodological steps is provided in Figure 2. While our aim was explicitly not to conduct a systematic review of the literature, our methodology might still be further improved in future studies deploying this embedded review approach.

In what follows, we discuss 13 specific ethical challenges related to the development of data-driven DSTs for cancer care that arose from our embedded review of the 4D PICTURE project. While we started from general themes (eg, ethical aspects regarding *semantic bias* and *fairness* in relation to text mining patient experiences to develop a conversation tool), we reframed the themes as challenges to provide a slightly more action-oriented overview that can serve as an agenda for embedding ethics into the project (a challenge is then “preserving meaning in the data and including underrepresented groups”). We note that there is no agreed-upon definition of what an ethical challenge is [10], but for the purpose of this paper, we defined it as follows: “an ethical challenge occurs when one does not know how to behave and act in the best way” [11]. To aid readability in our reporting of ethical challenges, in the text of this paper, we combined several 4D PICTURE work packages into more general headings (eg, combining “project management, dissemination and ethics,” which are 3 separate work packages in the 4D PICTURE project, into 1 section), but a full overview of the project’s work packages and related challenges is presented in Table 1.

**Table 1:** Ethical challenges related to the work packages (WPs) in the 4D PICTURE (Producing Improved Cancer Outcomes Through User-Centered Research) project. Descriptions of each WP’s main objectives were taken from the 4D PICTURE project’s website. It should be noted that challenge 13 is relevant across all WPs, although extra responsibility may be put on the project leads in WP 1.

WP	Main objectives	Ethical challenges
WP 2—modeling	The overall objective of WP 2 is to develop algorithms that provide predictions of outcomes for individual patients for each relevant treatment option for 3 major cancers. The algorithms will be translated into decision support tools and included in the MetroMap as developed in WP 4.	Avoiding biased outcomes due to poor data quality (challenge 1) Understanding how ethical values are built into models (challenge 2)
WP 3—text mining	The main aim of WP 3 is to use co-design methods to investigate the experiences of patients with cancer and their significant others, drawing upon novel text mining and qualitative analysis methodologies to improve outcomes for patients with cancer and their families and enhance the quality of a conversation tool to be used by citizens, patients, and clinicians across Europe.	Preserving meaning in the data and including underrepresented groups (challenge 3) Protecting the privacy of the data of patients with cancer in the public domain (challenge 4)
WP 4—design	The primary task of WP 4 is the redesign of care paths applying the service design method to experience a more individualized and personalized care path with the inclusion of innovative prognostic and conversational tools.	Incorporating shared decision-making and death into the care path design (challenge 5) Visualizing care paths responsibly through design justice (challenge 6)
WP 5—practice and WP 6—policy	In WP 5, researchers will evaluate MetroMapping in its entirety using mixed methods designs as well as evaluate the decision support tools to be developed. WP 6 aims to finalize the MetroMapping methodology, assess the generalizability of the methodology and decision support tools of MetroMapping, and provide guidance to policy makers about MetroMapping.	Reflecting on good criteria for (cost-effectiveness) evaluation (challenge 7) Balancing the adoption of technology with other values (challenge 8) Anticipating techno-moral change and developing new ethical frameworks (challenge 9)
WP 1—coordination and WP 8—dissemination	WP 1 entails all aspects of the coordination, management, and progress monitoring of the project. The task of WP 8 is to disseminate project information and results of the research and innovation activities to key stakeholders through various channels and enable access to decision support tools to patients with cancer, their significant others, clinicians, and the public.	Integrating patients’ experiential knowledge to avoid epistemic injustice (challenge 10) Negotiating shared knowledge in the trading zone between disciplines (challenge 11)
WP 7—ethics	WP 7 is a cross-cutting, integrative WP that establishes an embedded ethics approach within the 4D PICTURE project. It collaborates with all WPs and aims to ensure that ethical and social aspects of the planned tools and implications of their use are considered from the very start of their interdisciplinary development.	Establishing the position of the embedded ethicist (challenge 12)
All	—^a^	Ensuring psychological safety in research teams (challenge 13)

^a^Not applicable.

## Results

### Overview

Through our embedded review of ethics in the 4D PICTURE project, we identified 13 key ethical challenges related to the development of DSTs and the redesign of care paths for more personalized cancer care. These challenges are discussed in sections related to the various parts of this project: the prognostic model development; text mining of patient experiences to develop a conversation tool; the innovative design method of *MetroMapping* to develop a personal care path navigator; the evaluation of the MetroMap and integrated DSTs; and project management, dissemination, and ethics. An overview of how the identified ethical challenges relate to the 8 specific (but interrelated) work packages in the 4D PICTURE project, along with those work packages’ main objectives, is provided in Table 1.

### Prognostic Models for Individual Treatment Outcomes

#### Overview

One of the aims of the 4D PICTURE project is to develop models that predict individual patient outcomes for each relevant treatment option for breast cancer, melanoma, and prostate cancer. These prognostic algorithms will be translated into DSTs. Outcome measures will not only be related to survival but also operationalize different aspects of quality of life (eg, side effects or sexual well-being) per treatment option so that patients can make an informed treatment decision based on their personal circumstances and values. However, ethical aspects should be taken into account when developing such prognostic models. Hereafter, we discuss 2 key ethical challenges: the risk of data bias and the need for awareness that societal values are always built into models.

#### Ethical Challenge 1: Avoiding Biased Outcomes Due to Poor Data Quality

The accuracy of prognostic models depends heavily on the quality and representativeness of the data used. Data bias (ie, results being skewed because of unjust inaccuracies in the data used for modeling) can cause harm to individuals and increase existing inequities in society [12-14]. Biased outcomes can arise due to false assumptions incorporated into data collection, inconsistent definitions, small sample sizes, reproduction of societal trends influencing the data, and the underrepresentation of (minority) groups in datasets [15-17]. In the 4D PICTURE project, bias may be introduced as the input data for the prognostic models come from multiple European countries and from international randomized clinical trials and meta-analyses and, as such, may not be generalizable to, for instance, small patient groups or smaller European countries that lack representation in these datasets (let alone to countries in the Global South, although that is also not the aim of the 4D PICTURE project). For example, data may be predominantly derived from patients with European heritage, and ethnic minority groups may be underrepresented in the data, possibly leading the model to draw misguided conclusions about these patients [17-20]. In addition, models trained on a dataset in one setting often do not perform well in other settings [21]. To mitigate the impact of data bias, the 4D PICTURE researchers will weigh the quality and generalizability of the different data sources.

However, it may turn out that, for specific patient populations, the level of evidence is so low that a prediction tool may cause more confusion than clarity about treatment options for the patient [22,23]. If the evidence is relatively uncertain, does that mean that the clinician can refrain from presenting the patient with the model’s outcome to prevent confusion? We find that clinicians should evaluate the usefulness of the model’s output for each patient before consultation. However, open questions remain. At what level of certainty is the clinician obliged to share results with the patient to fulfill duties of openness and transparency? In addition, should the use of a prediction model, if available, be incorporated into the standard workflow or rather as an optional step that requires the patient’s informed consent before inputting data? Or would providing patients with information and choice lead to an undesirable redistribution of responsibility in which the clinician shifts the burden of making difficult decisions to the patient [24]? These issues call for ethical reflection and careful consideration of how clinical practice changes when prognostic models are introduced in a specific cancer care setting.

#### Ethical Challenge 2: Understanding How Ethical Values Are Built Into Models

While prognostic models are sometimes thought of as objective calculators, in reality, no algorithm is perfect. Models are not value free and will always have certain undesirable outcomes even when they merely output a prediction without coupling it to advice [25]. Namely, if two developers create a model based on the same database, the resulting models will be different because certain choices about the functioning of the model are made by the developer (eg, whether to accept more false negatives vs more false positives). Clinical risk prediction models are often programmed to prioritize *sensitivity* (fewer false negatives) over *specificity* (fewer false positives) because this reflects the existing tendency of human clinicians to better be safe than sorry (ie, to prefer the risk of overtreatment to the risk of undertreatment) [15]. In the same way, the selection of outcome measures is loaded with values. Overall survival, recurrence-free survival, and progression-free survival are commonly accepted outcome measures. However, not every outcome may be equally important to each patient.

Thus, prognostic models may be perceived within clinical practice as more neutral, objective, certain, and reliable than they actually are. This is akin to what some scholars have called the *automation bias* of humans regarding automated systems—people tend to rely too heavily on automated systems such as AI technologies and forget the cultural and ideological choices that were made during the development of that system [26,27]. It is important for developers and users to understand that these choices often reflect existing societal bias and inequalities. Moreover, developers of prognostic models may be faced with difficult moral dilemmas in which no right solution can be readily modeled [28]. If there is then a lack of ethics guidance, this can lead to arbitrary decision-making based on technical features such as computing power [29]. Even if developers themselves are very much aware of the limitations, uncertainties, value preferences, and subjective cutoff points of a model, these may not be immediately clear to the clinicians, patients, and policy makers who use the information generated by the model. Prognostic models are value laden and bring about ethical questions, but these are sometimes not recognized as such. Acceptability of certain choices during model development should be based on ethical reasoning and arguments, preferably in consultation with ethicists and a diverse range of stakeholders. The 4D PICTURE project offers a unique possibility to jointly study and discuss the ethical values built into the planned prediction models and their potential influence on actual cancer care practices.

This also includes discussion about *which variables* to include in predictive algorithms. For model developers, the accuracy of predictions has always been most important. However, depending on the intended use, a more accurate model may sometimes reproduce or even increase unfair inequalities in society. Imagine a model that calculates the expected quality of life after the treatment of a stroke. We know that, after a stroke, the average quality of life is much lower in neighborhoods where many people are of a lower socioeconomic status than in neighborhoods where most people are of a high socioeconomic status [30]. Including the postal code in the model might result in more accurate predictions of posttreatment quality of life. The question is then whether it is ethically justified to include postal code as a variable. The answer to this depends not only on the question of whether the variable makes the model more accurate but also on the intended use of the model. The reasoning is different for a model intended to allocate resources in a way that improves care in disadvantaged neighborhoods versus a model intended to decide who to give stroke treatment and for whom stroke treatment is not cost-effective because of a low predicted quality of life after the treatment. The latter use is problematic as it further unfairly disadvantages the already disadvantaged given that the low quality of life likely relates to factors outside individuals’ control, such as housing or health literacy. Whether to include variables that might affect treatment options for disadvantaged or protected groups, such as postal code but also gender, ethnicity, disability, BMI, and smoking or diabetes status, is a question that is relevant in the context of cancer care and in need of more interdisciplinary research and ethical reasoning.

### Text Mining of Experiences of Patients With Cancer to Develop a Conversation Tool

#### Overview

Another objective of the 4D PICTURE project is to conduct text mining analyses of *big* data on the experiences of patients with cancer to develop a conversation tool and obtain input for care path redesign. An interdisciplinary approach will be used that combines the strengths of AI tools (ie, text mining and natural language processing techniques), corpus linguistics, and qualitative (narrative) research to efficiently convert the stories of people with cancer and their significant others into usable knowledge. Key ethical challenges revolve around the risk of societal bias and loss of meaning in the data, as well as privacy and ownership questions regarding data scraped from online platforms.

#### Ethical Challenge 3: Preserving Meaning in the Data and Including Underrepresented Groups

In developing an algorithm for text mining of public forums, there is a risk of reproducing existing biases in society and even exacerbating them. Research has shown that applying machine learning to ordinary human language results in humanlike semantic biases. Namely, *text corpora* or language datasets contain imprints of our societal biases toward gender or race [31]. A well-known example is Google Translate’s translation of job descriptions in gender-neutral languages that do not differentiate between *he* and *she* into English—until recently, this produced only biased sentences such as “she takes care of children” and “he is a lawyer.” Moreover, subtle differences in meaning might be lost when transforming written experience into classifiable input for a conversation tool. If the eventual 4D conversation tool does not perform as well for each patient due to engrained societal biases or the loss of meaning in language processing, this can have a substantial impact in terms of fairness. For instance, if the tool works suboptimally for a certain minority group, this would not only be unfair but might also serve as a *microaggression*. A simple example of a microaggression is when an automatic soap dispenser cannot identify dark skin, which serves as a small (and unnecessary) reminder for people that their skin is not the *default* skin. Added up, these very small and unexpected daily encounters can truly affect a person’s sense of belonging in a society [32]. Thus, it is important that metaphors used for capturing the experience of patients with cancer in the 4D PICTURE project are accurate and reflect not only the metaphors of dominant cultures but also the cultural languages of different minority groups.

#### Ethical Challenge 4: Protecting the Privacy of the Data of Patients With Cancer in the Public Domain

Data mining of public posts on web forums is not without ethical issues. A recent review [33] mentioned the following aspects: the privacy policies of public forums sometimes lack transparency [34]; users are not always aware of privacy settings or lack the digital skills to manage them according to their preferences [35]; even if users are aware that their posts are public, this does not mean that they agree with their posts being reused for just any purpose [36]; commercial use in particular (which is not part of the 4D PICTURE project) is deemed inappropriate by users [37-39], whereas reuse for the greater good is more accepted; and some users are even willing to put privacy concerns aside for public benefit [40]. There are 2 general issues that stand out. First, the use of social media posts for research brings about privacy risks and, in particular, the potential for reidentification. There is a risk of reidentification both on an individual and group level (eg, identifying a minority group). Patients fear that reidentification may lead to identity theft; could have consequences for employment and pension eligibility; and could lead to increased insurance costs and the use of their data for financial gain, social discomfort, or stigmatization in clinical settings or the community [33,41]. Although there is clear consensus about ensuring anonymity regarding research that scrapes data from web forums, this is not always possible in practice. This first concern is only indirectly related to 4D PICTURE project, which will not use the data beyond its primary research aims, let alone to reidentify individual patients, but it does highlight the need for good data protection and security measures to protect the sets of data *scraped* from public forums.

Second, it is debated in the literature whether posts on public forums and social media should be considered as being part of the public or private domain given that notions of public and private are changing [40]. Legally, these posts are in the public domain, and no informed consent is needed regarding reuse, so many researchers do not ask for consent for scraping data from online forums [42-44]. However, citizens may have other intuitions about this. The distinction between *private* and *public* is probably too broad to reflect their moral intuitions about data ownership. The discrepancy between regulations and the views of citizens could lead to public outrage and less trust in science in general. Some ethicists have also argued that data may only be collected from social media platforms after explicit consent from the data subject [45]. It may be helpful in this setting to view privacy in terms of *contextual integrity*, a concept by Nissenbaum [46] based on the spheres of justice by Walzer [47]—simply put, contextual integrity says that privacy means something different in an airport security area than in a kindergarten. This is recognized by patients, who regard health data research as part of the (highly regulated) medical sphere and want their data to stay within that sphere [48], but what happens to contextual integrity when patients post their own health information on social media platforms? In the 4D PICTURE project, researchers will further explore these questions together with patient representatives to develop ethical guidance regarding the use of social media data for the project.

### MetroMapping to Develop a Personal Care Path Navigator

#### Overview

In addition to the development of prognostic and conversation tools, the 4D PICTURE project aims to visualize the care trajectory for melanoma, breast cancer, and prostate cancer in 3 countries (the Netherlands, Spain, and Denmark). The new methodology of *MetroMapping*, which involves collaborative meetings with health care workers, is used to create a comprehensive visualization of the care trajectory for each specific cancer, representing various treatment options as metro lines [49]. The resulting MetroMap consists of different layers in addition to the metro line itself, which represents the overall structure of the care trajectory; other layers incorporate patient experiences at various points in this trajectory (eg, highlighting aspects such as magnetic resonance imaging being perceived as frightening) or provide information about the environment (eg, parking options or quiet routes within the hospital to accommodate heightened sensitivity to noise and smell after chemotherapy). The function of the MetroMap is to locate potential for improvements and *redesign* the care paths of people with cancer where needed. In the 4D PICTURE project, the prognostic models and conversation tools will be integrated into the MetroMap to also develop a personal care path navigator for each patient that serves as a decision aid in shared decision-making (SDM). There are 2 main ethical topics relevant here: first, SDM and, second, design justice and the influence of choice architecture.

#### Ethical Challenge 5: Incorporating SDM and Death Into the Care Path Design

The concept of SDM is increasingly recognized as an important aspect of personalized care, and it lies at the core of the 4D PICTURE project. The goal of SDM is to engage in conversations about relevant treatment options and the patient’s values and preferences to arrive at a shared treatment decision aligned with their wishes [50]. In the 4D PICTURE project, the MetroMap design includes designated points labeled as *SDM moments* (indicated by stars). These moments signify important moments (eg, when test results are available) to allow for discussions between health care providers and patients regarding preferred treatment options. However, within actual care practice, SDM is complex and scattered along the care path, extending beyond a specific SDM moment occurring at a specific time point. Patients often struggle to express their values or preferences, necessitating support such as probing questions, multiple conversations, or sources of inspiration to determine what matters most to them in a given situation [51]. Some authors have argued that SDM runs the risk of putting too much responsibility on the patients and leaving patients “abandoned to their autonomy,” as O’Neill [52] has said. However, the everyday reality of SDM in actual care practices shows that autonomy has a relational nature—there is an interdependence among patients, their support networks, and their health care providers [53,54]. Thus, when performed well, SDM seems compatible with a notion of autonomy that does not put too much responsibility on patients. However, of note is that patients differ in their preferences for how care is delivered (eg, in the number and duration of consultations in which treatment decisions need to be made [55]), so it is important to keep in mind that one patient may not represent the entire patient population in their preferences for SDM. Moreover, and this is a more general point, social determinants of health—such as economic status, social vulnerability, and access to resources—shape patients’ capacity to engage in SDM, and health care systems should address these broader determinants to better support diverse patient needs related to SDM [56].

Another challenging aspect of the personal care path is the death of the patient. Death and mortality raise profound ethical questions, balancing values such as human dignity, patient autonomy, relieving pain, quality of life, and balancing the interests of an individual person within their network. As death carries cultural and social significance and ethical considerations vary across systems and belief systems, it is a notoriously sensitive topic in health care. Both health care workers and the systems of health care are mainly geared toward curing diseases and prolonging life—until relatively recently, clinicians were not taught to discuss end-of-life issues [57]. During the last decades, palliative care and advance care planning have been developed and professionalized [58], but how to incorporate end of life into the cancer care MetroMap is still an open question. Is there an end point? How should death be visualized? These are difficult questions and answers that may also differ by patient, country, and culture [59,60]. Studies show that prognostic and conversation tools may be helpful for discussing end-of-life care [61], but their development should be accompanied by reflection on the experience of the patient.

#### Ethical Challenge 6: Visualizing Care Paths Responsibly Through Design Justice

In the 4D PICTURE project, service design methods will be developed and used by designers to help create the MetroMap. This methodology may give rise to ethical questions, for instance, because the design researchers will work with people with different levels of power (medical team vs patient and family) and need to be aware of sensitivities and vulnerabilities and also because there is a risk of bias on the part of the researcher who leads this process [62]. Moreover, we noted previously that technologies themselves (eg, algorithms) are not neutral but are packed with values [63]. The same is true for the cancer care path design as the way in which it is set up spatially may affect how information is perceived. The lens of *design justice* draws attention to this and shows how design enables or encourages certain actions while excluding or discouraging others. These design aspects are known as *affordances*—properties of an object that suggest possible actions that users can take (eg, a button affords pushing). In the context of the 4D PICTURE MetroMap, questions arise regarding its affordances and disaffordances. For example, if the map relies heavily on color, it may not be accessible to clinicians with color blindness. Identifying such issues within the design process is crucial to ensure that the MetroMap is inclusive. Thus, design justice brings awareness to often unconscious design decisions and seeks to rectify historical and systemic injustices perpetuated by design decisions (eg, a bridge that is too low for a public bus to pass under, allowing only car owners to take a certain road [64]). Design justice challenges power dynamics within design, advocating for the redistribution of resources and opportunities to address social, economic, and environmental disparities [63].

**Figure 3:**
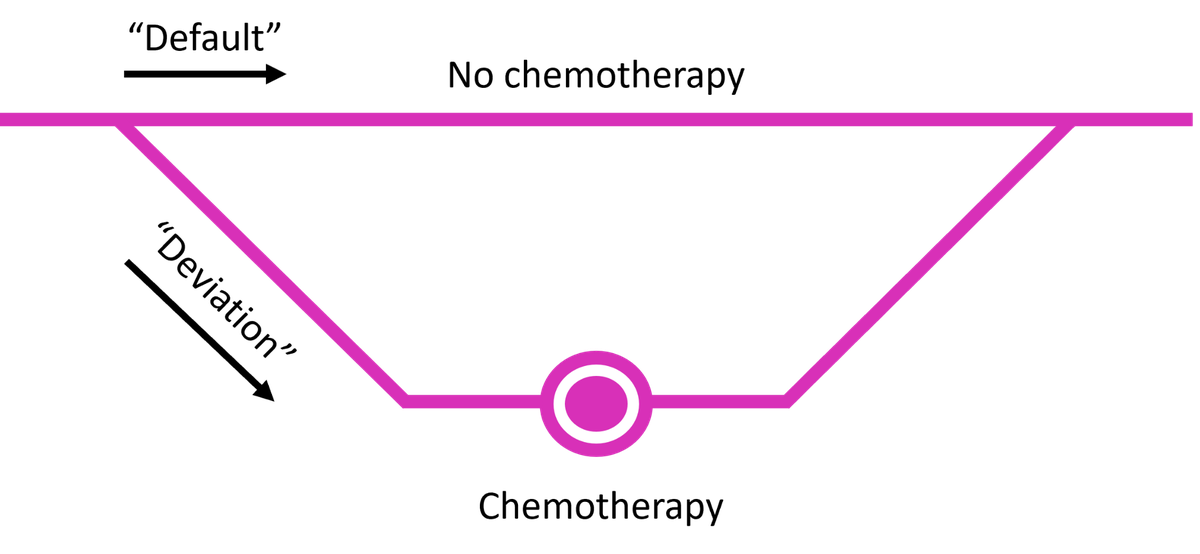
Choice architecture—visualization of the default and deviated decision paths.

An important part of design justice in the 4D PICTURE project is the impact of what has been dubbed *choice architecture* (ie, the number of options presented simultaneously, or their order, influence which information is best retained by viewers and which choice is finally made [64-68]). To illustrate, it makes a difference whether treatment options are presented as “no treatment vs. treatment” or “no treatment vs. treatment option 1 vs. treatment option 2” because people divide their attention equally in considering all options [66]. The visualization of *default* and *deviated* decision paths (Figure 3) can also have a large effect on which choice is made [69]. There is little evidence from health care on this topic, but cartography research shows that the linearity of map routes matters in route choices made by travelers [70]. The effects of choice architecture are so strong that it works even if decision makers are aware of the mechanism [71]. One paper about default options in oncology concludes that further experimental studies are needed to select which default options successfully change behavior [72], and to this we add that such investigation should include consideration of design justice.

Some ethics scholars suggest that choices should be presented in the least directive way, but others have argued for using conscious choice architecture to stimulate unpopular but beneficial choices (ie, nudging) [73]. For example, some types of localized prostate cancer generally present as slow-growing tumors, and active surveillance of progression while withholding or postponing treatment is a beneficial option in this case as treatment has a significant chance of producing complications [65]. However, medicine is biased toward acting rather than waiting, leading some clinicians to present patients more strongly with the different treatment options than with the possibility of active surveillance. In this example, a MetroMap might need to be designed as a choice between active surveillance and treatment first and then as a choice between treatments. MetroMap designers should deliberate regarding which effect they wish the MetroMap to have on decisions and how this is best achieved and underpin the acceptability of this choice with medical-ethical arguments. Although exerting as little influence as possible is preferable in most cases, there are situations in which some nudging by using ordering effects or default options can be argued to be justified.

### Evaluation and Development of Guidance for Policy Makers

#### Overview

The 4D PICTURE project also contains an evaluation of the MetroMapping design methodology, the MetroMap itself, and the prognostic model and conversation tool, as well as a cost-effectiveness evaluation and an assessment of the generalizability of the methods and developed tools. Moreover, the project will develop guidance for policy makers about MetroMapping using a framework that helps understand the nonadoption; abandonment; and challenges to the scale-up, spread, and sustainability (NASSS) of health technologies by professionals or patients [74]. With regard to the evaluation and policy aspects of the 4D PICTURE project, the following ethical topics are relevant: reflection on the criteria for evaluation, balancing ethics with technology adoption, and techno-moral change.

#### Ethical Challenge 7: Reflecting on Good Criteria for (Cost-Effectiveness) Evaluation

In the 4D PICTURE project, questionnaires among patients, significant others, and clinicians will be used to compare between the original and redesigned care paths and evaluate the developed tools. As is good practice within quantitative evaluation studies and expected by funding agencies, the criteria based on which the redesigned care paths will be evaluated were defined in advance. Researchers should consider, together with patient representatives, which evaluation method and criteria fit their project best. The same is true for cost-effectiveness analyses. The ultimate goal of such analyses is to help health care decision makers choose between competing alternatives based on some predetermined measure of economic efficiency, such as cost per life saved, cost per year of life saved, or positive net benefits. Just like we saw with prognostic models, an important ethical aspect of cost-effectiveness analyses is that they “expose...hidden assumptions, and require explicit judgements to be made about which ethical position is appropriate in a particular policy context” [75]. Should cost-effectiveness analyses be used at all, or is it inherently unjust to compare costs between persons [76]? Such ethical questions and specific dilemmas regarding cost-effectiveness have been discussed extensively in the medical ethics literature, but further work is still needed to see how existing arguments apply to the novel setting of data-driven and personalized cancer care.

#### Ethical Challenge 8: Balancing the Adoption of Technology With Other Values

Research shows that many innovations are eventually not adopted [77]. In the 4D PICTURE project, the NASSS framework is used to study how the developed tools can be designed to promote adoption and avoid wasting resources. However, strategies that increase the adoption potential of health innovations can simultaneously pose ethical dilemmas. An example is that of a research laboratory that developed care robots, where the adoption by health care workers was a major issue (eg, some nurses put the robots in a closet because they found them annoying) [78]. The researchers discovered that building gender stereotypes into the robots contributed to their acceptability and adoptability by health care workers. For instance, if they developed a robot that was interpreted as female, the tone of voice was expected to be much more modest and less authoritative than that of robots that were interpreted as male. This poses an ethical dilemma: what is the right thing to do [79]—contributing to acceptability by repeating gender stereotypes into the design or countering gender stereotypes? Ethical reflection with stakeholders is needed on how to balance adoption with other values. This can take the form of structured methods such as moral case deliberation [80] as well as structural collaboration between researchers and groups of patients and the publics, which is discussed under ethical challenge 10.

#### Ethical Challenge 9: Anticipating Techno-Moral Change and Developing New Ethical Frameworks

Innovations are almost never used in practice as intended, and they often produce unintended, unforeseen, and sometimes even counterproductive consequences—in other words, “things bite back” [81]. A simple example is how, when more highways are built to reduce traffic jams, more people will take their cars, so no reduction in traffic jams is realized. Often, new technologies or other innovations influence our concepts and conceptions of a good life [82,83]. Think of the introduction of the contraceptive pill that contributed to women’s emancipation or of blood sugar measurement devices that influence the way in which patients with diabetes relate to their bodies [84]. This process of technology and ethics codeveloping has been referred to as *techno-moral* change [83]. Techno-moral change is notoriously hard to research or predict and may complicate the evaluations and policy development planned in the 4D PICTURE project and similar health technology projects. In addition, the 4D PICTURE project itself may contribute to techno-moral change through the development of prognostic models and the conversation tool and the redesign of care paths—these may influence patients’ and clinicians’ moral routines in unexpected ways or create new moral dilemmas. Possible techno-moral changes should be anticipated (eg, through qualitative interviews about the expectations of stakeholders or through ethnographic observation studies) during the research phase instead so that they may be acted upon in time.

A timely example of techno-moral change is the incorporation of sustainability into evaluation frameworks and to take the costs, harms, and burdens of health care technology with respect to the environment into account. Health care has always been a system with a significant carbon footprint due to the many single-use products, and now data are also becoming an important factor [85]. The increasing data storage and analysis possibilities bring about new moral questions. For instance, is it proportionate to slightly reduce a certain health risk using a method that puts a considerably larger burden on the environment? However, we currently lack ethical vocabulary, frameworks, and research on this topic. The connection and trade-offs between health care and planetary health need to be further studied, and this important aspect will be included in the 4D PICTURE project despite not being in the original plan. For instance, to prime researchers about the topic, the authors of this paper organized an interactive session about environmental sustainability during the latest project consortium meeting and asked each work package to present in the next consortium meeting how they will address sustainability in their work.

### Project Management, Dissemination, and Ethics

#### Overview

Similar to most large research projects, the 4D PICTURE project has several *work packages* focused on aspects bordering the science at the project’s core—the coordination, management, and progress monitoring of the project, as well as the dissemination of findings and the embedding of ethics throughout the project. Although it is less obvious, even these work packages can give rise to ethical issues. We identified the following issues that are relevant for the 4D PICTURE project and other research projects on data-driven care: epistemic injustice, the *trading zone*, the position of the embedded ethicist, and psychological safety.

#### Ethical Challenge 10: Integrating Patients’ Experiential Knowledge to Avoid Epistemic Injustice

The 4D PICTURE project integrates experiential knowledge from patient and public involvement (PPI) boards in all participating countries aiming to align care paths with patients’ needs. However, bridging experiential and academic knowledge presents challenges. How to translate the information shared by the PPI boards into usable knowledge for the research group? What to do with contradictions or differences of opinion [86,87]? A helpful concept is that of epistemic injustice or, in other words, knowledge-related injustice [88]. This encompasses testimonial and hermeneutical injustices [89]. Testimonial injustice arises when dismissing patient experiences as unreliable, emotional, or irrelevant, potentially leading to a loss of confidence that causes patients to stay silent [90]. Hermeneutical injustice arises when patients lack the language or concepts to articulate their experiences [90]. This occurs because these concepts have not been developed yet, because patients do not have access to them, or because the concepts are not recognized by clinicians as the dominant group. In the 4D PICTURE project, the conversation tool is intended to allow patients to express their experiences and bridge this hermeneutical gap between patients and professionals. Awareness of the concept of epistemic injustice may help the researchers notice instances in which it may play a role and search for strategies to minimize epistemic injustice during the research process. Strategies include conveying patient contributions through stories or alternative mediums such as visual art, films, or metaphors [90,91]. Researchers must also undertake “role, emotion, and relationship work,” which involves switching between different roles (eg, researcher, facilitator, advocate, relation manager, or coffee maker), handling loaded emotions with care and empathy (ie, rather than distancing oneself from the subject), and fostering relationships [92]. Awareness of these strategies helps minimize epistemic injustice, ensuring that patients’ voices are valued and respected in the research process. A final note is that power dynamics leading to epistemic injustice are influenced by existing structural inequalities regarding race or socioeconomic class, for instance. In the 4D PICTURE project, the ethicists and other researchers will work with the PPI boards on appropriate ways (eg, payment of the PPI boards) to alleviate the harmful effects of such structural concerns to help ensure that all voices are heard equally.

#### Ethical Challenge 11: Negotiating Shared Knowledge in “the Trading Zone” Between Disciplines

Often, professionals from different disciplines use different concepts; ascribe different significance to objects or phenomena; use different conceptual frameworks; and may also have different value systems, accountability rules, and quality indicators. The same is true for clinicians and patients in the cancer care trajectory—clinicians’ choices and priorities may conflict with the perspectives of patients (eg, streamlining and speeding up the care path vs preferring more time to deliberate treatment options between multiple consultations [55]). Understanding how to guide collaborations between different scientific disciplines and with patient representatives in the 4D PICTURE project can be based on the metaphor of a *trading zone*, which is often used to study multidisciplinary scientific collaborations [93]. In the *trading zone*, a shared understanding between the different disciplines and different types of (experiential) knowledge should be negotiated. This *trading* can be facilitated by an agent who is familiar with >1 discipline. Sometimes, this could be a nurse; in other cases, it can be an embedded ethicist. To do this effectively and fairly, it is important to have awareness of power differences and implicit value frameworks. Are all relevant voices heard and valued equally [94]? Who has a say in the structure and processes of collaboration? Whose knowledge counts (see the aforementioned concept of *epistemic injustice*)?

To avoid one perspective overshadowing another, collaborators should think about how to handle differences of opinion in meetings and how to ensure that all participants make sufficiently equal contributions. Strategies to divide power equally in the trading zone can consist of making the differences in vocabulary, systems of recognition, and value systems explicit; making use of *boundary objects* that facilitate exchange between multiple worlds [95]; develop meeting structures that stimulate an explicit deliberation; and decide in advance which agents in a project are best placed to facilitate collaboration in the trading zone (this is not always the project lead). Of note is that the trading zone metaphor is relevant not only during the conduct of research but also in the dissemination phase. Dissemination is in itself an ethical imperative, especially when the research was publicly funded, but also comes with ethical sensitivities [96]. Collaborative efforts between different types of partners (eg, clinician researchers, developers, and patient representatives) can help facilitate the *trading* or sharing of knowledge in a way that is valuable for reaching different stakeholders. As such, dissemination can help combat epistemic injustice as well as *demystify* scientific concepts and hypes such as those surrounding AI [97]. Dissemination should always be planned so that the outputs are contextualized and sensitive to the experiences of the group under study (in our case, patients with cancer) [98].

#### Ethical Challenge 12: Establishing the Position of the Embedded Ethicist

Ethicists are embedded in the 4D PICTURE project to conduct this literature review and several empirical studies about ethical issues, as well as to provide support with ad hoc ethical issues by joining scientific meetings and providing internal trainings. In bioethics, there have been long-standing debates about how empirical data relate to normative reasoning and about the role of the ethicist in health research [99]. Should an ethicist be a critical, distant outsider or be part of the research practice? We find that a more *embedded* role is called for right now. Take the example of AI—while several organizations have developed general principles for AI in health care, the ethical difficulties lie in applying these principles and making trade-offs in actual research practices. Embedded ethics can fill this gap between intentions and actions by engaging in practical and relational work on ethics within a specific research project or development process [7]. However, there are also some risks and disadvantages to embedded ethics. Namely, as the ethicist’s normative analysis is so close to research practice, there is a risk that more fundamental ethical questions will not be discussed and researched. An example is the growing influence of *big tech* on our health care systems—as research ethics committees have only focused on topics such as privacy in individual research projects and specific tools to be developed, they lack an ethical framework to meaningfully weigh the broader collaboration with industry. Ethicists can sometimes secure power instead of challenging it because “they locate the source of the problem in individuals or technical systems instead of acknowledging structural power differences and working structurally towards dismantling them” [100]. In certain cases, there is a risk of this leading to *ethics washing* or lip service to industry [7]. Thus, in large interdisciplinary health research projects, one should not forget to consider “the ethics of the ethics work package.” Moreover, we find that ethics should be recognized as a shared responsibility—the embedded ethicists can *sensitize* other consortium members to the ethical aspects but cannot be solely responsible for the normative assessments of the research and the tools being developed.

#### Ethical Challenge 13: Ensuring Psychological Safety in Research Teams

Finally, an underappreciated ethical aspect of research is psychological safety, which is defined as “the belief that one will not be punished or humiliated for speaking up with ideas, questions, concerns, or mistakes, and the team is safe for interpersonal risk taking” [101]. Safe environments are those where it does not feel risky to express one’s thoughts, doubts, questions, and errors. As such, psychological safety is a key condition for high-performance research teams as this safe environment promotes takings risks as well as reporting mistakes and learning from them. Psychological safety also influences the degree to which people speak up about (research) misconduct and promotes open discussions about *grey areas* or so-called *questionable research practices* such as cutting corners due to time pressure [102]. In the same vein, it is a precondition for opening up about moral dilemmas in research and development. Strategies for promoting psychological safety include encouraging vulnerability, active listening, appreciating diverse perspectives, promoting a culture of feedback and learning, establishing clear expectations, celebrating experimentation, treating mistakes as learning opportunities, providing tools and training for effective conflict resolution, using metrics to regularly assess psychological safety levels, and leadership actively endorsing psychological safety [101]. Of course, in large projects, the level of psychological safety differs by collaborating partner, but it can still be influenced by the project and work package leads.

## Discussion

In this paper, we have described relevant ethical challenges that should be considered when developing data-driven DSTs for more personalized cancer care. We based our review on the European 4D PICTURE research project, and as such, this paper is the first review grounded explicitly in the *Embedded Ethics* approach [7]. Using a collaborative and iterative methodology helped provide a broad overview of the ethics of this heterogeneous and interdisciplinary project. This overview serves as starting point for developing actionable guidance for the project and potentially beyond, as well as for further empirical and theoretical ethics research and future collaborations with developers, clinicians, researchers, and the PPI boards in the 4D PICTURE project. As such, we add to discussions in previous, more systematic literature reviews about data-driven DSTs in health care, which have mostly highlighted general, high-level ethical principles of respect for patient autonomy, prevention of harm, fairness, explicability, and privacy [103], as well as professional autonomy, bias and justice, and explicability [104]. Another study applied an ethical framework on AI-guided clinical decision support and used case examples to illustrate key issues of accountability and transparency, the potential for group harm, efficiency of health care, and conflicts between roles and responsibilities [105]. Such examples are insightful but provide less detail than a review of a complete research project. The iterative process of extracting themes by moving between key papers and discussions with the consortium helped us describe more specific examples of the universal themes in the literature and uncover additional challenges unrelated to the technical and medical core of the project. As such, our embedded review highlights ethical aspects previously neglected in the digital health ethics literature and zooms in on real-world challenges in an ongoing project.

Several identified ethical challenges (challenges 1-4) were related to the data and algorithms needed to develop data-driven DSTs in oncology. Prominent issues were indeed bias and privacy, which have been extensively described in previous literature reviews, yet our analysis gave rise to more specific, real-world questions for further research and advice (eg, “what are the arguments for including or excluding postal code in a prognostic model for cancer treatment outcome?” or “should posts on online patient fora be considered part of the public domain or is consent needed to use them for the development of conversation tools in oncology?”). Other topics (challenges 5 and 6) revolved around the specific design methodology used in the 4D PICTURE project. Questions arose about *design justice* that have not been previously addressed in the context of DSTs for health care. Furthermore, we described various ethical aspects related to broader policy issues in health care (challenges 7-9). Lysaght et al [105] have previously mentioned the cost-effectiveness of data-driven DSTs and financial sustainability of health care systems as important ethical issues, and we have added that environmental sustainability is also a timely ethical consideration—currently, there is a lack of knowledge on how to apply sustainability in practice and how to balance it with competing values.

Finally, various challenges that we described in this paper do not relate directly to the DST as the outcome of the 4D PICTURE project but rather to the research process itself (eg, to the project management or results dissemination). We highlighted the importance of epistemic justice in the collaboration with patients and between different disciplines, questioned the role of the embedded ethicist who may not be fully independent themselves, and called for attention to psychological safety (challenges 10-13). The latter topic is increasingly discussed in various sectors of work, including health care, but is generally not an agenda point for international consortia even though project leaders seem to have an important role model function. Therefore, a key outcome of this embedded ethics review is that researchers and leaders in medical data-driven research projects should not forget the ethical challenges of the work that surrounds the scientific core of the project.

Our methodology is novel but not without limitations. The literature search was not systematic, so the results are not exhaustive, and the identification of ethical challenges was to a certain extent subjective and colored by the preferences and knowledge of the ethicists that conducted it. We addressed this by conducting a *member check* with 4D PICTURE researchers, but we propose that, in future projects, a check by an external ethicist could serve as additional validation. In addition, the definition of *ethical challenges* could be developed together with participants (in our case, the researchers in the 4D PICTURE project) to avoid variation and achieve clarity in the analysis and interpretation [10]. Moreover, new issues may come up after the publication of this paper as the project is still evolving. While these will be addressed in annual documents shared within the consortium (a sort of *rolling review*), this paper is not as dynamic or adaptable. Further thought is needed on how to better match the publication of ethics research with the developments in medical and data science and the possibility for techno-moral change (eg, should it be possible to update papers after publication in an academic journal to ensure that they remain up-to-date and relevant?). Finally, a general limitation of embedded ethics research is inherently tied to its main strength of providing actionable recommendations within a specific project—namely, ethicists should ensure not to lose sight of the broader structural and systemic issues underlying the particular questions discussed within the scope of a project. For instance, the focus on the 4D PICTURE project limited our discussions on data bias to *the* European context, if that even exists, whereas a huge concern is *data poverty* in low- and middle-income countries that may have fewer means to digitalize health care systems [106]. Another example relates to the more structural socioeconomic inequalities that influence digital inclusion efforts, which cannot be *solved* within applied research projects focused on developing digital tools [107]. We need to consider, in future work, how embedded ethicists can put such structural issues on the agenda when their work is tied to specific ethics work packages in highly delineated projects.

In conclusion, this viewpoint shows that a lot may be gained when ethics is embedded into a project from the start. Analysis of existing literature was deepened, and findings were made more actionable through the iterative collaboration between ethicists and other researchers in a large research consortium. For instance, the work on this embedded ethics review led to further collaboration with the design researchers in the 4D PICTURE project to pre-emptively reflect on ethical challenges that may arise during their fieldwork of *shadowing* patients to analyze oncology service design [62], thus laying the groundwork for responsible visualization and redesign of care paths (challenge 6) while ensuring that patients and their voices are respected in the research process (challenge 10). We suggest that ethicists working in interdisciplinary projects should not automatically opt for systematic or scoping reviews of a single question but rather consider whether a more applied *Embedded Ethics* review strategy might sometimes better fit their practical and theoretical aims. Ethics often formulates abstract, high-level principles, and in this paper, we have shown how these can be operationalized in practice, in particular when designing and developing data-driven support tools for improving cancer care. Taking on board the identified challenges and recommendations will help ensure that data-driven innovations in oncology are developed in an appropriate and patient-centered manner.

## Appendix

Multimedia Appendix 1 4D PICTURE Collaborator Authorship.

## Abbreviations

AI: artificial intelligence
DST: decision support tool
NASSS: nonadoption; abandonment; and challenges to the scale-up, spread, and sustainability
PICTURE: Producing Improved Cancer Outcomes Through User-Centered Research
PPI: patient and public involvement
SDM: shared decision-making
