# Supplementary material for: Ethical Design of Data-Driven Decision Support Tools for Improving Cancer Care: Embedded Ethics Review of the 4D PICTURE Project
Source: JMIR Cancer. 2025 Apr 10;11:e65566. doi: 10.2196/65566 (PMC12022531; doi:10.2196/65566)
Supplement: Multimedia Appendix 1 [file cancer_v11i1e65566_app1.docx]

**Supplementary File 1: 4D PICTURE Collaborator Authorship**

The scientific members of the 4D PICTURE consortium who are not authors on this manuscript. Names and affiliations are as follows:

Daisy Monika Lal¹, Yufeng Liu², Uwe Siebert³, Yassin Engelberts⁴, Paul Rayson⁵, Marie Kremer⁶, Karina Dahl Steffensen⁷, Suzan Verberne⁸, Anne Stiggelbout⁹, Sheila Payne¹⁰, Gaby Sroczynski³, Amara Callistus Nwosu¹¹, Monique J. Roobol¹², Tamara Wit⁹, Lára R. Hallsson³, Therese Pross¹³, Ellen Kapiteijn¹⁴, Toby Hackmann⁹,¹⁵, Michel W.J.M. Wouters⁹,¹⁶, Teresa Puértolas¹⁷, Roberto Pazo-Cid¹⁷, Juan Lao Romera¹⁷, Cornelis Verhoef¹⁸, Ewout Steyerberg¹⁹, Lifeng Han⁹,⁸, Ignacio López-Forniés², Liesbeth C. de Wreede²¹, Marleen Kunneman⁹, Jasper Brands²², Sander Puts²³, Iñaki Alvarez Busto¹⁷, Carlos Romero-Piqueras²⁴, Judith Rietjens⁴,²⁵, Paula Melo Signerez²⁵, Claudia Cruz Oliveira⁴, Dirk Snelders²⁵, Erik van Mulligen²⁶, Chris Bangma¹², Barbara Peric²⁷, Erika Renedo Illarregi²⁰, Lionne D.F. Venderbos¹², Elfi M. Verheul⁴, Aina Johnsson²⁸

¹ School of Computing and Communications, Lancaster University, Lancaster, United Kingdom
² Department of Linguistics and English Language, Lancaster University, Lancaster, United Kingdom
³ Institute of Public Health, Medical Decision Making and Health Technology Assessment, UMIT TIROL – University for Health Sciences and Technology, Hall in Tirol, Austria
⁴ Department of Public Health, Erasmus MC, University Medical Center Rotterdam, Rotterdam, the Netherlands
⁵ UCREL, School of Computing and Communications, Lancaster University, Lancaster, United Kingdom
⁶ Lab for Social Design, Design School Kolding, Kolding, Denmark
⁷ Center for Shared Decision Making Vejle/Lillebaelt University Hospital of Southern Denmark; Institute of Regional Health Research, Faculty of Health Sciences, University of Southern Denmark, Odense, Denmark
⁸ Leiden Institute of Advanced Computer Science, Leiden University, Leiden, the Netherlands
⁹ Medical Decision Making, Department of Biomedical Data Sciences, Leiden University Medical Center, Leiden, the Netherlands
¹⁰ International Observatory on End of Life Care, Lancaster University, Lancaster, United Kingdom
¹¹ Marie Curie Hospice Liverpool, Liverpool University Hospitals NHS Foundation Trust, Liverpool, United Kingdom
¹² Department of Urology, Cancer Institute, Erasmus MC, University Medical Center Rotterdam, Rotterdam, the Netherlands
¹³ Department of Gynecology with Breast Center, Charité Universitätsmedizin Berlin, Berlin, Germany
¹⁴ Department of Medical Oncology, Leiden University Medical Center, Leiden, the Netherlands
¹⁵ Medical Statistics, Department of Biomedical Data Sciences, Leiden University Medical Center, Leiden, the Netherlands
¹⁶ Dutch Institute for Clinical Auditing, Leiden, the Netherlands
¹⁷ Department of Medical Oncology, Miguel Servet University Hospital, Universidad de Zaragoza, Zaragoza, Spain; Instituto Investigación Sanitaria Aragón (IIS-A); Hospital Universitario Miguel Servet, Zaragoza, Spain
¹⁸ Department of Surgery, Cancer Institute, Erasmus MC, University Medical Center Rotterdam, Rotterdam, The Netherlands
¹⁹ Julius Center, University Medical Center Utrecht, Utrecht, the Netherlands
²⁰ Department of Engineering Design and Manufacturing, University of Zaragoza, Zaragoza, Spain
²¹ Medical Statistics, Department of Biomedical Data Sciences, Leiden University Medical Center, Leiden, the Netherlands
²² Panton, Deventer, The Netherlands
²³ Department of Radiation Oncology (MAASTRO), GROW School of Oncology and Developmental Biology, Maastricht University Medical Center, Maastricht, The Netherlands
²⁴ Fractal Strategy, Zaragoza, Spain
²⁵ Department of Design, Organisation and Strategy, Faculty of Industrial Design Engineering, Delft University of Technology, Delft, the Netherlands
²⁶ Department of Medical Informatics, Erasmus MC, University Medical Center Rotterdam, Rotterdam, the Netherlands
²⁷ Institute of Oncology Ljubljana, Medical faculty Ljubljana, University of Ljubljana, Ljubljana, Slovenia
²⁸ Department of Oncology-Pathology, Karolinska Institute, Stockholm, Sweden
